# Effectivness of Nebulized Budesonide for COPD Exacerbation Management in Emergency Department; a Randomized Clinical Trial

**Published:** 2020-10-28

**Authors:** Mehrad Aghili, Elnaz Vahidi, Narges Mohammadrezaei, Tina Mirrajei, Atefeh Abedini

**Affiliations:** 1 Department of Emergency Medicine, Shariati Hospital, Tehran University of Medical Sciences, Tehran, Iran.; 2 Prehospital and Hospital Emergency Research Center, Tehran University of Medical Sciences, Tehran, Iran.; 3 Chronic Respiratory Diseases Research Center, National Research Institute of Tuberculosis and Lung, Masih Daneshvari Hospital, Shahid Beheshti University of Medical Sciences.

**Keywords:** Budesonide, Drug Administration Routes, Emergency Service, Hospital, Nebulizers and Vaporizers, Peak Expiratory Flow Rate, Prednisolone, Pulmonary Disease, Chronic Obstructive

## Abstract

**Introduction:**

Nebulized budesonide has been long used in chronic obstructive pulmonary disease (COPD) exacerbation. This study aimed to compare the effectiveness of nebulized budesonide (NB) versus oral prednisolone (OP) in increasing peak expiratory flow rate (PEFR) of COPD patients in emergency department (ED).

**Methods:**

Patients with COPD exacerbation, referring to ED were enrolled in this randomized trial study. In the first group, NB 0.5 mg every 30 minutes till three doses, placebo tablet, and standard treatment was administered. In the second group, nebulized normal saline, OP tablet 50 mg, and standard treatment were administered. Patients’ demographic data, vital signs, PEFR, venous blood gas (VBG) analysis, disposition, and patient and physician satisfaction were all collected and compared between the two groups.

**Results:**

43 patients in the NB group and 41 patients in the OP group were evaluated. The two groups had similar age (p=0.544) and gender (p=0.984) distribution, duration of illness (p=0.458), and baseline PEFR (p=0.400). 12 and 24 hours after treatment, significant increase in PEFR in the NB and OP groups were observed (p=0.032 and 0.008; respectively). The upward trend of PEFR in NB group was significantly better than that of OP group during 24 hours of treatment (p=0.005). Vital signs and VBG results showed no significant differences between the two groups during the studied time interval.

**Conclusion:**

NB, compared to OP, could more effectively increase PEFR and ameliorate disease severity of patients with COPD exacerbation at 12 and 24 hours after treatment in ED.

## Introduction

American Thoracic Society/European Respiratory Society (ATS/ERS) guidelines defined chronic obstructive pulmonary disease (COPD) as an irreversible and progressive airflow limitation. It is now the fourth leading cause of death in the world (1). COPD causes a significant disease burden based on its severity and clinical course (2). 

Systemic corticosteroids (SCS) and inhaled corticosteroids (ICS) have been endorsed by medical societies in the treatment of acute exacerbation of chronic COPD since a long time ago (3, 4). Corticosteroid consumption is recommended in addition to bronchodilator, oxygen, and antibiotic treatment in moderate to severe COPD (5). Despite its proven benefits, there are still concerns regarding acute and chronic adverse effects of SCS (6, 7). Hyperglycemia, myopathy, osteoporosis, and adrenal gland suppression are mentioned as significant concerns (8). In this regard, ICS seems to be a more exciting option due to having fewer undesirable effects.

Nebulized corticosteroid (NCS) has been safely used as a substitute for ICS since the last decade (8, 9). Its consumption leads to changes in gas exchange parameters, which is believed to be due to its anti-inflammatory effects causing a decrease in obstruction and airway resistance (9). Some preliminary data suggest that NCS has similar efficacy to SCS in management of COPD cases, but still, further studies are needed, especially in the emergency setting. A review article in 2012 concluded that NCS could be used as a valuable alternative for ICS in asthma and COPD (10, 11). It also recommended additional research to confirm this promising role in the acute setting.

Nebulized budesonide (NB) is one of the most popular NCS that has been used in both Asthma and COPD, since a long time ago. 

This study aimed to compare the effectiveness of NB versus oral prednisolone (OP) in increasing peak expiratory flow rate (PEFR) of COPD patients in emergency department (ED).

## Methods

***Study design and setting***

This double-blind, randomized clinical trial was performed in 3 general university hospitals (Dr. Shariati, Imam Khomeini and Sina hospitals) during 6 months in 2019. Patients with COPD exacerbation, referring to ED were enrolled. In the first group, NB 0.5 mg every 30 minutes till three doses, placebo tablet, and standard treatment and in the second group, nebulized normal saline, OP tablet 50 mg, and standard treatment were administered. PEFR and VBG analysis of the two groups were compared during the first 24 hours after treatment.

The study was approved by the ethics committee of Tehran University of Medical Sciences (Ethics code: IR.TUMS.MEDICINE.REC.1397.108; IRCT ID: IRCT20180523039800N1). The manuscript adheres to the “Reporting of noninferiority and equivalence randomized trials: extension of the CONSORT 2010 statement”. 

***Participants***

All patients older than 18 years with moderate to severe COPD exacerbation, referring to the EDs of the mentioned hospitals were enrolled in our study. The eligibility criterion was having an established (pre-existing) diagnosis of COPD, not the new probable cases. The diagnosis of COPD exacerbation was established by an emergency physician (EP) as defined by ATS/ERS (1, 4). The exclusion criteria were: prior history of asthma, allergic rhinitis, interstitial lung disease, atopy, diabetes mellitus, hypertension, previous history of SCS or ICS consumption in the previous month, loss of consciousness, acute respiratory failure needing intubation and mechanical intubation, psychologic disorder, language barrier, or unwillingness to participate in our study.

***Interventions***

Sampels were enrolled by block randomization (randon permuted block) of 4 (allocation ratio 1:1). Randomisation was performed using unmarked, ordered, sealed envelopes. Patients were randomly allocated to either the first group (nebulized budesonide (NB) 0.5 mg every 30 minutes till three doses (Pulmicort Respules® manufactured by AstraZeneca LP), placebo tablet, and standard treatment) or the second group (nebulized normal saline every 30 min till three doses, OP tablet 50 mg (manufactured by Iran Hormone), and standard treatment). Standard treatment included: oxygen administration with the goal of oxygen saturation (SPO_2_) > 90%, frequently inhaled β-agonist (salbutamol) via the metered-dose inhaler (MDI) device, anticholinergic (ipratropium bromide) via MDI device (4 puffs every 20 min during the first hour and then continued by 2-4 puffs every 6 hours), and appropriate antibiotic selection. The acute treatment was started within 1 hour after patient admission. Budesonide or normal saline was administered via A3 Complete Omron Compressor Nebulizer. The color and shape of studied drugs were the same in both groups.

The chief investigator generated the random allocation sequence, and assigned and enrolled participants in each group. The triage nurse administered the specified drug with the proper method, route and dose of administration a previously prepared. The EP on each shift evaluated the patient, assessed outcoms and filled the checklist. EP, triage nurse, and patient were all blinded to the study.

***Data gathering***

Demographic data, patient satisfaction, and disposition were recorded. Vital signs, venous blood gas (VBG) parameters (partial pressure of oxygen (PO2), partial pressure of carbon dioxide (PCO2) and potential hydrogen (pH)), and PEFR were assessed and documented at 0, 30, 60 min and 3, 6, 12 and 24 hours after admission. Measurement of arterial blood gas is not the mainstay of ED evaluation in COPD anymore. Response to therapy can be easily monitored sufficiently by the patient’s clinical status, pulse oximetry, and VBG, if necessary.

Patient and physician satisfaction were evaluated based on the objective response to treatment on a scale from 0 (the worst) to 3 (the best).

***Primary and secondary endpoints***

Our primary endpoint was comparing PEFR before and after the treatment between the two groups. Our secondary endpoints were comparisons of VBG parameters, vital signs, patient satisfaction, and disposition between the two groups.

***Statistical analysis***

All patients fulfilling the inclusion critera during the studied 6-month interval were enrolled in this study. A sample size of 40 patients in each group was calculated by considering; α: 0.05, β: 0.20, θ1-θ2: 0.1 (0.1 Liter difference in FEV1 between the two treatment regimens) based on reference (8), r:1 and δ: 0.1. 

To compare baseline values, Pearson’s chi-square test (for qualitative variables) and Independent t-test (for quantitative variables) were used. For comparison of changes in parameters between and within groups, the repeated measures analysis of variance (ANOVA) test was used. The data were analyzed using SPSS version 22.0 (SPSS Inc.). The mean values and 95% confidence intervals (Cis) in each group were calculated. A p-value of <0.05 was considered significant.

## Results

***Baseline characteristics of studied patients***

**Figure 1:**
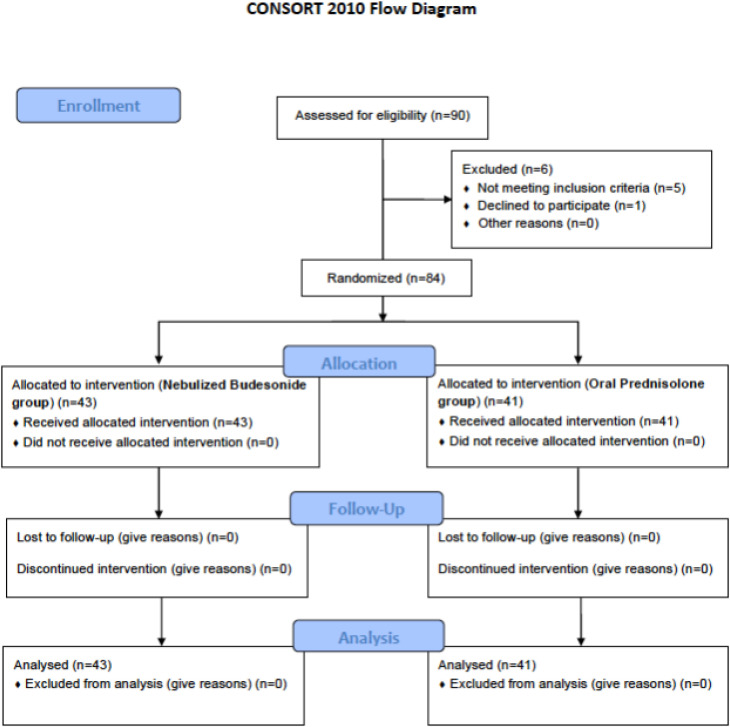
Flow diagram of the study

Finally, 43 patients in the first group (NB) and 41 patients in the second group (OP) were included (figure 1). Table 1 compares the patients’ baseline characteristics between groups. The two groups had similar age (p=0.544) and gender (p =0.984) distribution, duration of illness (p =0.458), and baseline PEFR (p =0.400) and VBG analysis (p>0.05).

***Outcomes***

Table 2 compares the vital signs and VBG parameters of patients at different times of evaluation between the two groups. PEFR 12 (p = 0.032) and 24 (p = 0.008) hours after treatment were the only variables that were significantly different between groups.

**PEFR**

**Figure 2:**
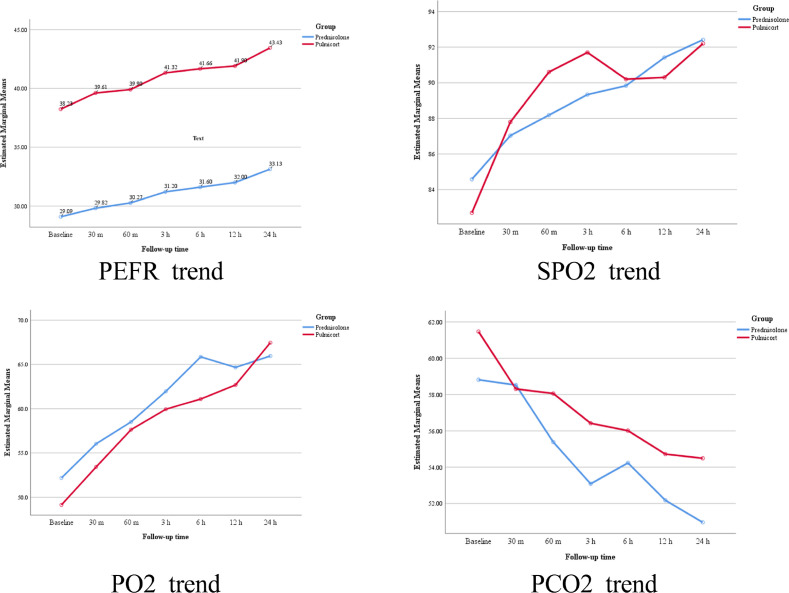
Peak expiratory flow rate (PEFR), Oxygen saturation (SPO2), Partial pressure of oxygen (PO2), and Partial pressure of carbon dioxide (PCO2) changes during the studied time interval in both oral prednisolone (OP) and nebulized budesonide (NB) groups

PEFR had significantly increased from the baseline within each group 24 hours after the treatment (p= 0.024 in the NB group and 0.001 in the OP group). Comparing the two groups’ data showed that PEFRs at 12 and 24 hours after the treatment had improved more significantly in the NB compared to the OP group (p= 0.032 and 0.008; respectively). PEFR assessment during the studied time interval disclosed that the upward trend of PEFR was more significant in the NB compared to the OP group (p= 0.005) (figure 2). 

**SPO**_2_

SPO_2_ results at 24 hours after the treatment were increased from the baseline in both groups. However, this difference was significant only in the OP group (p= 0.108 in the NB group and 0.001 in the OP group). A comparison of the SPO_2_ trend showed no significant difference between the two groups during the studied time interval (p= 0.887) (figure 2).

**PO**_2_

PO_2_ results at 24 hours after the treatment were increased from the baseline within both groups (p= 0.001 in both groups). A comparison of the PO_2_ trend showed no significant difference between the two groups during the studied time interval (p= 0.574) (figure 2).

**PCO**_2_

PCO_2_ results at 24 hours after the treatment were decreased from the baseline in both groups. However, this difference was significant only in the OP group (p= 0.073 in the NB group and 0.015 in the OP group). A comparison of the PCO_2_ trend showed no significant difference between the two groups during the studied time interval (p= 0.619) (figure 2). 

**Patient and physician satisfaction**

Patient and physician satisfaction with the treatment process were evaluated and graded from the worst (0) to the best (3). Patient satisfaction had a mean score of 1.81±1.01 in the NB group and 1.88±0.88 in the OP group (p= 0.876). Physician satisfaction had a mean score of 1.82±1.06 in the NB group and 1.69±0.97 in the OP group (p= 0.643). 

The ultimate disposition of patients was evaluated in 27 patients in the NB group and 24 cases in the OP group. It was determined that 18 cases (66.6%) in the NB group needed hospital admission longer than a day, and 9 cases (33.4%) were discharged after 24 hours. In the OP group, 23 patients (95.8%) were admitted to the hospital ward, and only one patient (4.2%) was discharged after 24 hours (p= 0.012).

## Discussion

In the present study, it was determined that during the acute phase of COPD exacerbation in ED, NB was more effective than OP at 12 and 24 hours. It reduced COPD severity (based on PEFR) at 12 and 24 hours after the treatment, more than OP. We observed the upward trend of PEFR, and we found that this increasing trend was more significant in the NB group compared to the OP group. VBG parameters were all changed for the better during disease recovery. However, their trend was not significantly different between the two groups. Our results also stated that more patients in the OP group were admitted to the hospital and needed more definite care after 24 hours, while in the NB group, more patients were discharged after a day.

Gunen et al. in 2007, studied severe COPD patients (12). They administered the standard treatment (bronchodilator) alone to group 1, OP+bronchodilator to group 2, and NB+bronchodilator to group 3. OP was administered as 40 mg intravenous solution, and NB was administered every 6 hours at a dose of 0.5 mg/2 ml. Cases were hospitalized for more than ten days and received the exact dose mentioned. They evaluated patients for a longer time during follow-up, and they found that at 24 and 72 hours and seven days, much better results and recovery were observed in groups 2 and 3. They also showed that after ten days, more patients in group 1 were discharged from the hospital compared to the other two groups. Finally, they concluded that in the acute phase, the recovery rates with regards to spirometry and arterial blood gas parameters did not differ between the groups utilizing some form of corticosteroid. However, recovery rates in these groups were significantly better than that of the group receiving only bronchodilator treatment.

Maltais et al. in 2002, performed a multicenter, double-blind, randomized placebo-controlled trial on 199 patients with COPD exacerbation (8). They compared NB, OP, and placebo in 3 different groups. 2 mg NB was administered every 6 hours in group 1, 30 mg OP tablet was administered every 12 hours in group 2, and finally, the specified placebo was ordered in group 3. All patients received the standard treatment. Patients were evaluated from baseline to 72 hours after drug administration, and they had a follow-up of 10 days. Their results determined that the use of NB and OP was associated with a faster rate of improvement in spirometry compared with placebo. Less adverse effects were observed in group 1. They suggested that NB might be an alternative to OP for the treatment of acute nonacidotic exacerbation of COPD.

Mirici et al. in 2003, analyzed 44 patients with COPD exacerbation (9). They either received 40 mg parenteral prednisolone or 4 mg NB every 12 hours. They evaluated PEFR and arterial blood gas parameters at 30 min, 6, 12, 24, and 48 hours and also day 10. They demonstrated that there were significant increases in PEFR, PO_2_, and SPO_2_ values within the groups. No significant differences were observed between the groups about the percentage of changes in PEFR, PO_2_, and SPO_2_ values during the entire period of assessment. 

ICSs are the mainstay of anti-inflammatory treatment in asthma and COPD. Melani et al. in 2012, reviewed 16 clinical trials on asthma and COPD and concluded that NCS is currently a valid alternative to inhalers in asthma and COPD. They recommended further research to confirm the promising role of NCS in acute settings (13).

Guade et al. in 2012 reviewed eight previous studies comparing NCS vs. SCS (10). In all of their included studies, NB had been used in acute exacerbation of COPD in different dosages, and it had been compared with either parental or oral SCS. All the studies had confirmed the clinical efficacy of NB to be of similar extent to that of SCS. They also recommended more extensive studies to be designed and done.

Rodrigo, in 2006, published a review article in the CHEST journal and evaluated the rapid effects of ICS in acute asthma (11). Their data showed that ICS could have early beneficial effects (even 1 to 2 h), especially when used in multiple frequent doses and administered in short time intervals. The nongenomic effect was said to be a possible contributor to the clinical effects of corticosteroids. 

The current study confirmed that NB, when administered at frequent doses (0.5 mg) at shorter time intervals (every 30 min) up to 3 doses, could more significantly increase COPD patients’ PEFR at 12 and 24 hours compared to OP. This method of administration showed us that we could discharge more patients in a shorter time.

**Table 1:** Comparison of patients’ baseline characteristics between nebulized budesonide (NB) and oral prednisolone (OP) groups

**Variable**	**NB** **(n = 43)**	**OP (n = 41)**	**P-value**
**Age (years)**			
Mean ± SD	64.47±10.53	66.11±10.22	0.544
**Gender N (%)**			
Male	34 (79.0)	32 (78.0)	0.984
Female	9 (21.0)	9 (22.0)	
**Duration of disease (years)**			
Mean ± SD	6.40 ± 4.91	5.56 ± 3.30	0.458
**Smoking duration** **(years)**			
Mean ± SD	32.08 ± 13.73	26.50 ± 12.69	0.135
**PEFR (%)**			
Mean ± SD	35.18±12.83	32.67±9.76	0.400
**SPO2 (%)**			
Mean ± SD	84.97±9.96	83.75±9.79	0.633
**Pulse rate (/minutes)**			
Mean ± SD	92.27±14.42	100.89±16.40	0.363
**Respiratoty rate (/minutes)**			
Mean ± SD	22.88±5.50	23.93±7.60	0.535
**PO2 (mmHg)**			
Mean ± SD	45.59±13.22	46.86±14.80	0.726
**PCO2 (mmHg)**			
Mean ± SD	56.49±13.72	54.72±9.13	0.622
**pH**			
Mean ± SD	7.35±0.06	7.34±0.05	0.460

Standard deviation (SD), Peak expiratory flow rate (PEFR), Oxygen saturation (SPO2), Partial pressure of oxygen (PO2) (Normal range: 30-40), Partial pressure of carbon dioxide (PCO2) (Normal range: 41-51), Potential Hydrogen (PH) (Normal range: 7.31-7.41).

**Table 2:** Comparison of vital signs and venus blood gas (VBG) parameters between nebulized budesonide (NB) and oral prednisolone (OP) groups

**P-value**	**OP (n = 41)**		**NB** **(n = 43)**		**Time** **(Hour)**	**Variable **
	**95%CI**	**Mean±SD**	**95%CI**	**Mean±SD**		
0.400	20.59-59.94	32.67±9.76	18.33-75.0	35.18±12.83	0	**PEFR** **(%)**
0.349	23.15-65.42	34.36±11.03	18.33-75.0	37.33±13.17	0.5	
0.346	24.07-67.29	35.65±11.55	17.50-80.77	38.91±14.34	1	
0.458	25.0-67.29	37.31±11.73	18.33-76.92	40.02±14.43	3	
0.175	23.53-59.72	35.52±8.92	20.18-63.64	40.44±13.47	6	
**0.032**	24.0-48.0	33.58±7.32	24.0-61.0	42.09±12.0	12	
**0.008**	25.41-49.32	33.13±6.40	28.70-60.34	44.43±9.38	24	
0.460	7.23-7.49	7.34±0.05	7.27-7.48	7.35±0.06	0	**pH**
0.414	7.21-7.44	7.34±0.06	7.23-7.46	7.35±0.06	0.5	
0.288	7.10-7.55	7.35±0.08	7.29-7.48	7.36±0.05	1	
0.327	7.20-7.50	7.35±0.08	7.29-7.46	7.37±0.04	3	
0.844	7.29-7.44	7.37±0.04	7.24-7.44	7.37±0.04	6	
0.403	7.30-7.40	7.35±0.03	7.30-7.43	7.36±0.04	12	
0.539	7.27-7.41	7.34±0.03	7.30-7.40	7.35±0.03	24	
**P-value**	**Range**	**Mean±SD**	**Range**	**Mean±SD**	**Time**	**Variable**
0.633	50-96	83.75±9.79	52-96	84.97±9.96	0	**SPO2** **(%)**
0.654	70-98	89.48±6.11	68-99	90.22±6.56	0.5	
0.331	73-98	90.64±6.11	74-98	92.10±5.24	1	
0.594	75-97	91.76±5.22	78-98	92.52±4.98	3	
0.889	79-98	91.35±4.94	82-97	91.14±4.51	6	
0.533	80-98	91.40±4.34	78-97	90.17±5.81	12	
0.861	87-96	92.42±2.68	87-96	92.20±3.05	24	
0.363	62-130	100.89±16.40	70-127	92.27±14.42	0	**PR** **(/min)**
0.411	61-125	96.79±13.74	72-120	94.09±11.43	0.5	
0.484	65-127	93.30±13.77	73-118	91.03±10.67	1	
0.118	70-120	92.40±12.15	75-103	87.81±8.46	3	
0.414	78-116	91.30±9.83	88.0-108	88.95±8.33	6	
0.859	75-100	87.53±7.48	85-94	87.92±3.06	12	
0.740	78-97	86.92±6.36	80-95	86.10±4.70	24	
0.535	14-48	23.93±7.60	16-35	22.88±5.50	0	**RR** **(/min)**
0.541	13-40	22.43±6.05	15-33	21.60±4.43	0.5	
0.960	15-39	20.74±5.44	15-31	20.81±4.45	1	
0.765	14-30	19.48±4.12	15-32	19.85±4.74	3	
0.632	15-32	19.65±4.15	14-32	20.38±5.43	6	
0.446	15-30	19.27±3.47	16-28	20.33±3.65	12	
0.826	15-21	18.67±2.01	16-21	18.50±1.35	24	
0.726	15-91	46.86±14.80	20-74	45.59±13.22	0	**PO2** **(mmHg)**
0.138	34-84	54.73±12.63	22-84	49.08±15.53	0.5	
0.108	35-93	55.97±12.77	16-80	49.99±14.62	1	
0.230	31-85	55.18±12.51	25-72	50.68±13.66	3	
0.311	37-78	59.01±10.54	25-75	55.20±12.84	6	
0.436	39-80	63.50±9.80	21-78	59.15±17.64	12	
0.647	51-85	65.95±8.70	57-76	67.45±5.79	24	
0.622	24-88	54.72±9.13	29-89	56.49±13.72	0	**PCO2** **(mmHg)**
0.792	26-85	53.49±14.01	39-71	52.64±9.87	0.5	
0.695	31-85	51.50±12.17	31-76	52.72±11.14	1	
0.337	31-75	48.71±10.63	32-78	51.73±11.37	3	
0.662	39-73	51.46±10.10	37-71	52.94±11.12	6	
0.824	34-70	52.15±11.52	38-71	53.18±11.82	12	
0.453	32-65	50.96±10.52	40-71	54.49±11.07	24	

Data are presented as mean ± standard deviation with 95% confidence interval (CI) or range. Peak expiratory flow rate (PEFR), Oxygen saturation (SPO2), pulse rate (PR), Reapiratory rate (RR), Partial pressure of oxygen (PO2), Partial pressure of carbon dioxide (PCO2), Potential Hydrogen (pH).

## Limitations

The sample size is small so the generalizability of the results is one of our limitations. Further studies with larger sample size are required to determine the exact effect of NB on COPD exacerbation. Frequent assessment by PEFR and VBG was time-consuming, and some patients got tired of frequent assessments. We did not evaluate patients’ current medications or previous intensive care unit admissions (baseline disease burden).

## Conclusion:

NB was more effective than OP in increasing PEFR at 12 and 24 hours in COPD exacerbation and, in the acute phase, NB could ameliorate COPD severity more efficiently compared to OP. 
